# Neurobiological Alterations in Females With PTSD: A Systematic Review

**DOI:** 10.3389/fpsyt.2022.862476

**Published:** 2022-06-13

**Authors:** Elizabeth Eder-Moreau, Xi Zhu, Chana T. Fisch, Maja Bergman, Yuval Neria, Liat Helpman

**Affiliations:** ^1^New York State Psychiatric Institute, Columbia University Irving Medical Center, New York, NY, United States; ^2^Department of Psychiatry, Columbia University Irving Medical Center, New York, NY, United States; ^3^Department of Counseling and Human Development, Faculty of Education, University of Haifa, Haifa, Israel; ^4^Psychiatric Research Unit, Tel Aviv Medical Center, Tel Aviv, Israel

**Keywords:** PTSD, females, sex differences, neuroimaging, MRI

## Abstract

Most females experience at least one traumatic event in their lives, but not all develop PTSD. Despite considerable research, our understanding of the key factors that constitute risk for PTSD among females is limited. Previous research has largely focused on sex differences, neglecting within group comparisons, thereby obviating differences between females who do and do not develop PTSD following exposure to trauma. In this systematic review, we conducted a search for the extent of existing research utilizing magnetic resonance imaging (MRI) to examine neurobiological differences among females of all ages, with and without PTSD. Only studies of females who met full diagnostic criteria for PTSD were included. Fifty-six studies were selected and reviewed. We synthesized here findings from structural MRI (sMRI), functional MRI (fMRI), diffusion tensor imaging (DTI), and resting state functional connectivity (rs-FC MRI) studies, comparing females with and without PTSD. A range of biopsychosocial constructs that may leave females vulnerable to PTSD were discussed. First, the ways timing and type of exposure to trauma may impact PTSD risk were discussed. Second, the key role that cognitive and behavioral mechanisms may play in PTSD was described, including rumination, and deficient fear extinction. Third, the role of specific symptom patterns and common comorbidities in female-specific PTSD was described, as well as sex-specific implications on treatment and parenting outcomes. We concluded by identifying areas for future research, to address the need to better understand developmental aspects of brain alterations, the differential impact of trauma types and timing, the putative role of neuroendocrine system in neurobiology of PTSD among females, and the impact of social and cultural factors on neurobiology in females with PTSD.

## Introduction

Nearly 90% of adults in the U.S. report lifetime exposure to at least one potentially traumatizing event, while only 8.3% go on to develop posttraumatic stress disorder (1). Females report slightly lower rates of overall trauma exposure as compared to males, but are twice as likely to meet diagnostic criteria for PTSD (2). Females' proclivity to develop PTSD more frequently than males may be partially explained by greater exposure to specific types of trauma relative to males (e.g., intimate partner violence). However, such an explanation is too glib and fails to consider relevant biological (sex) and psychosocial (gender) substrates of PTSD in females that could provide further clarification as to why females develop PTSD at higher rates than males, and why some females develop PTSD while others do not.

Over the past decade, scholars have begun to examine neurobiological markers of PTSD more closely. A growing body of evidence reflects differential neurological and clinical manifestations of trauma exposure according to sex [e.g., (3)]. Females appear to exhibit specific symptom patterns that vary according to trauma type, timing, and duration (4). For example, adult female victims of severe abuse, particularly sexual abuse, may display higher rates of dissociation than victims of other types of trauma (5, 6). Varying rates of PTSD among females have also been related to the neuroendocrine system, wherein estradiol regulation through the hypothalamus has been identified as a protective factor against developing PTSD following a traumatic event (7–9). Thus, the likelihood that females develop PTSD following trauma exposure may partially dependent on the phase of the menstrual cycle at the time of exposure. The extent of a female's risk of experiencing symptoms of PTSD may also be associated with learned coping styles influenced by social and cultural factors in the environment.

Psychosocial and cultural factors influencing symptom severity in PTSD in females pertain to gender socialization, social support, and cultural definitions of self and femininity, and ascription to traditional gender norms (10–13). Researchers have long posited that females process emotional information differently from males and that there is significant within-group variability that may be attributed to the aforementioned constructs (14, 15). For example, females who ruminate more demonstrate less cognitive flexibility and are thus more vulnerable to negative psychological outcomes, including exacerbated PTSD symptoms (15, 16). Such coping styles may be learned as part of cultural socialization to gender, wherein socialized masculinity is associated with more problem-oriented coping styles and femininity with emotional coping (15). Some, such as rumination, have been associated with poor psychological outcomes and distinct neural correlates (17). Culture may also influence the adaptation of personality traits associated with poor neurological and psychological outcomes, such as neuroticism, found to have its own neural correlates (16). In sum, it is likely that females' distinct risk for PTSD and subsequent severity is comprised of social, environmental, and biological factors. Despite the evidence that such covariates might cause substantial within group variability, few authors have reviewed existing neuroimaging literature regarding the difference between trauma exposed females who do an do not develop PTSD.

Literature reviews examining the role of biological sex in PTSD highlight the influence of the human and animal female neuroendocrine system on PTSD symptoms, fear responses, and extinction [e.g., (8, 18, 19)]. They report mixed findings in the literature, noting that varied impacts of estradiol and progesterone on fear extinction and PTSD symptoms underscore the need for a greater understanding of differences among females with PTSD. Seligowski et al. (19) similarly make important contributions to the current body of literature, highlighting differences between neural correlates of PTSD in males and females while also emphasizing the need for greater understanding of differences among females. A review of neuroimaging findings specific to trauma exposed female samples with and without PTSD would complement this literature, and elucidate future directions for both between and within group comparisons of females with PTSD.

The goal of the present review is therefore to examine and summarize the extant body of research relevant to within-group neuroimaging exploration of females with a diagnosis of PTSD. We hope to clarify neurobiological symptoms of PTSD in females, their prognosis for recovery, and areas for future research. We refer to the female sex throughout the review in order to accurately portray the literature and associated findings for a number of reasons. First, sex, defined as a dichotomous variable stipulated at birth by biology and genetics, encompasses the underlying neural mechanisms of PTSD and the neuroendocrine system. Conversely, gender is a nonbinary, socially defined construct that influences concepts of femininity and masculinity (19). While gender undoubtedly influences PTSD outcomes, there is ample evidence that the influence of such psychosocial factors on the development of PTSD may vary by sex (19). Additionally, in many of the studies summarized participants' gender identity is not consistently reported in the literature, term “female” may be a more accurate description of the relevant literature. While we use the term female and mainly refer to biological differences between PTSD and trauma exposed controls in the aftermath of exposure to trauma, it is important to note that these differences are not necessarily innate only and may interact with environmental factors.

## Methods

Database searches in PubMed, PsychInfo, and EBSCOHost were conducted to find peer-reviewed scholarly works exploring neural mechanisms and brain morphology related to PTSD in females, elucidated by neural imaging through MRI. We did not limit the timeframe of publications to allow for breadth in results. Search criteria included female samples of who had undergone MRI scans related to PTSD. Search terms included “neuroimaging,” “sex differences,” “PTSD,” “women,” “females,” “MRI” and “fMRI.” The initial search returned 87 articles, the abstracts of which were subsequently screened to determine whether they met inclusion and exclusion criteria. Only studies that included MRI scans (sMRI, fMRI, rs-FC) of female samples who met the full diagnostic criteria for PTSD were included. No animal studies met inclusion criteria. Studies including male samples were included as long as there were within-group analyses among females. Relevant articles were imported into Covidence, a software system designed to assist in systematic literature reviews, and submitted to other reviewers for their opinion. Of the initial articles, 24 were deemed irrelevant, leaving a total of 63 articles for full-text review. Following the review of the full-text, ten additional studies were removed because they did not meet the inclusion criteria. Subsequent to the initial search, a complementary manual search was conducted to ensure that the authors captured the full breadth of literature on PTSD in females. An additional search was conducted using the keywords “pediatric PTSD,” “MRI,” and “females.” This search returned 18 articles, of which only three were eligible for review according to inclusion criteria. As such, a total of 56 studies were included, as shown in Table 1.

**Table 1:** List of studies included.

**References**	**Sample size**	**Sample mean age and SD when available**	**Trauma type**	**Trauma timing**	**Imaging type**
**Adult samples**					
Aupperle et al. (20)	14	40.07 (SD = 7.44)	IPV	Adulthood	fMRI: Cued anticipation task
Aupperle et al. (21)	22	34.60 (SD = 9.40)	IPV	Adulthood	fMRI: Stop/signal Task
Berman et al. (22)	62	25.21	Sexual assault	Adulthood	MRI, fMRI- rsFC
Berman et al. (23)	62	25.21	Sexual assault	Adulthood	MRI
Brown et al., (24)	70	32	Interpersonal violence	Adulthood	fMRI: Implicit emotional interference/conflict task
Brown et al. (16)	61	31.05	Interpersonal violence	Adulthood	fMRI: Emotion interference/conflict matching task, rsFC
Buchholz et al. (17)	39	31.33 (SD = 9.38)	Interpersonal violence	Adulthood	fMRI: Emotion interference task
Cisler et al. (25)	16	33.8 (SD = 10.8)	Interpersonal violence	Adulthood	fMRI: trauma memory recall task
Cisler et al., (26)	40	33.26	Interpersonal violence	Adulthood	fMRI: trauma memory recall task
Cisler et al., (25)	16	33.8 (SD = 10.8)	Interpersonal violence	Adulthood	fMRI: trauma memory recall task
Crombie et al. (27)	121	33.15	Interpersonal violence	Adulthood	MRI
Felmingham et al., (28)	86	Not indicated	Mixed (accidents and interpersonal violence)	Adulthood	fMRI: fear perception task
Fonzo et al. (29)	24	Not indicated	IPV	Adulthood	fMRI: emotional face-matching task, rsFC
Fonzo et al. (30)	33	Not indicated	IPV	Adulthood	fMRI: emotional face processing task, rsFC
Graziano et al., (31)	21	31.9 (11.04)	Mixed interpersonal violence	Adulthood	DTI
Graziano et al., (32)	78	31.45	Mixed interpersonal violence	Adulthood	DTI
Jovanovic et al. (33)	41	39.2	Mixed, specific type not indicated	Not indicated	fMRI: Go/No go task
Landre et al. (34)	34	24.8	Sexual abuse	Adulthood	MRI
Moser et al. (35)	35	33.49	Interpersonal violence	Adulthood	fMRI: separation task
Moser et al. (36)	48	33.6 (5.4)	Interpersonal violence	Adulthood	fMRI: separation task
Neumeister et al. (37)	36	26.47	Interpersonal violence	Adulthood	fMRI: trauma-related picture processing task
Neumeister et al. (38)	38	26.84	Interpersonal violence	Adulthood	fMRI: trauma related word-processing task
New et al. (39)	42	36.3	Sexual abuse	Adulthood	fMRI: explicit emotion regulation task
Philippi et al. (40)	71	31.93 (9.39)	Interpersonal violence	Adulthood	fMRI: Self-related impact coding task, rsFC
Privratsky et al., (41)	65	33.7 (9)	Not indicated	Not indicated	fMRI: fear conditioning and extinction task
Ross et al. (42)	29	31.17	Interpersonal violence	Adulthood	fMRI: reinforcement learning task
Sartin-Tarm et al. (43)	43	30.8 (8.2)	Interpersonal violence	Adulthood	fMRI: fear conditioning and extinction task
Satterthwaite et al. (44)	105	31.22	IPV	Adulthood	fMRI: rsFC
Schechter et al. (45)	45	26.18	Interpersonal violence	Adulthood	fMRI: maternal separation task
Schechter et al. (46)	59	34.2 (5.7)	Interpersonal violence	Adulthood	fMRI: maternal separation task
Simmons et al. (47)	30	35.73	IPV	Adulthood	fMRI: cued anticipation task
Simmons et al. (48)	30	34.58	IPV	Adulthood	fMRI: cued anticipation task
Stevens et al. (49)	40	38.4	Mixed	Adulthood	fMRI: emotion regulation fear and neutral face processing task
Strigo et al., (50)	38	35.62	IPV	Adulthood	fMRI: experimental pain paradigm task
Vatheuer et al. (51)	99	29.38	Not specified	Not specified	MRI
Weaver et al., (52)	19		Interpersonal violence	Adulthood	fMRI: emotional conflict task
Weaver et al., (53)	61	32.34	Interpersonal violence	Adulthood	fMRI: reward-punishment contingency task
Bluhm et al., (54)	32	38.53	Childhood maltreatment	Childhood	fMRI: Free thinking task
Chalavi et al., (55)	61	42	Childhood maltreatment, Adult IPV	Childhood	MRI
Chalavi et al., (5)	65	39.48	Childhood maltreatment	Childhood	MRI
Frewen et al. (56)	30	37.22 (7.00)	Childhood maltreatment, interpersonal violence	Childhood	fMRI: emotional imagery/numbing task
Frewen et al. (57)	44	30.86	Childhood maltreatment	Childhood	fMRI: visual-verbal self-other referential processing task
Kitayama et al., (58)	18	37.3	Childhood maltreatment	Childhood	MRI
Lebois et al. (59)	65	34.37 (12.2)	Childhood maltreatment	Childhood	fMRI: interference, masked faces, and rest tasks
Ludascher et al. (60)	25	28.38	Childhood maltreatment	Childhood	fMRI: Dissociation-script task
Sierk et al. (61)	42	40.12	Childhood maltreatment	Childhood	fMRI Diffusion MRI
Steuwe et al. (62)	32	32.06 (12.03)	Childhood maltreatment	Childhood	fMRI: eye contact task
Steuwe et al., (62)	32	32.06	Childhood maltreatment	Childhood	fMRI: eye contact task
Tomoda et al. (63)	37	19.75	Childhood sexual abuse	Childhood	MRI
Veer et al. (64)	24	27.5	Childhood maltreatment	childhood	MRI
**Child/adolescent samples**					
Cisler et al., (65)	34	13	Childhood maltreatment	Childhood/ Adolescence	fMRI: implicit threat processing
DeBellis and Keshavan (66)	183	11.72	Childhood maltreatment	Childhood/ Adolescence	MRI
Klabunde et al. (67)	59	13.9	Interpersonal violence	Childhood	MRI
Letkiewicz et al. (68)	60	15.3	Interpersonal violence	Adolescence	fMRI: reinforcement learning task
Ross et al., (69)^**^	253	14.7 (1.9)	Childhood maltreatment	Adolescence	MRI

*fMRI, functional Magnetic Resonance Imaging; MRI, Magnetic Resonance Imaging; IPV, Intimate Partner Violence; DTI, Diffusion Tensor Imaging*.

** *Included adolescents and adults*.

## Results

In this literature review, we present putative factors impacting neurobiological and clinical presentations of PTSD in females. We review the role of key external determinants including trauma timing and trauma type, intrapersonal considerations and neural correlates that place females particularly at risk of developing PTSD, and conclude with the ways that these may culminate in the clinical presentations of PTSD in females. We then address the impacts PTSD has on both mothers' abilities to relate to their children, the neural substrates of mothers with PTSD, and the subsequent effects that has on their children. Finally, we end with a discussion of treatment outcomes among females with PTSD.

### The Role of Trauma Characteristics in Neurobiological Presentation

#### Trauma Timing

Trauma that occurs early in life severely influences brain development (70). The extent and location of the impact is modulated by the timing, type, and duration of the traumatic exposure during childhood (4, 68, 71). Because male and female brains mature differently due to sex differences endogenous to the neuroendocrine system, it is important to understand the effects of childhood trauma on the brain of females. One of the types of childhood maltreatment studied most frequently in the PTSD literature en masse is childhood sexual abuse (CSA) (66, 72). Surprisingly, there were few neuroimaging studies that met the inclusion criteria for our review. This section of the review presents the immediate and the long-term neurobiological impacts of CSA in females with PTSD documented in the neuroimaging literature.

**Figure 1:**
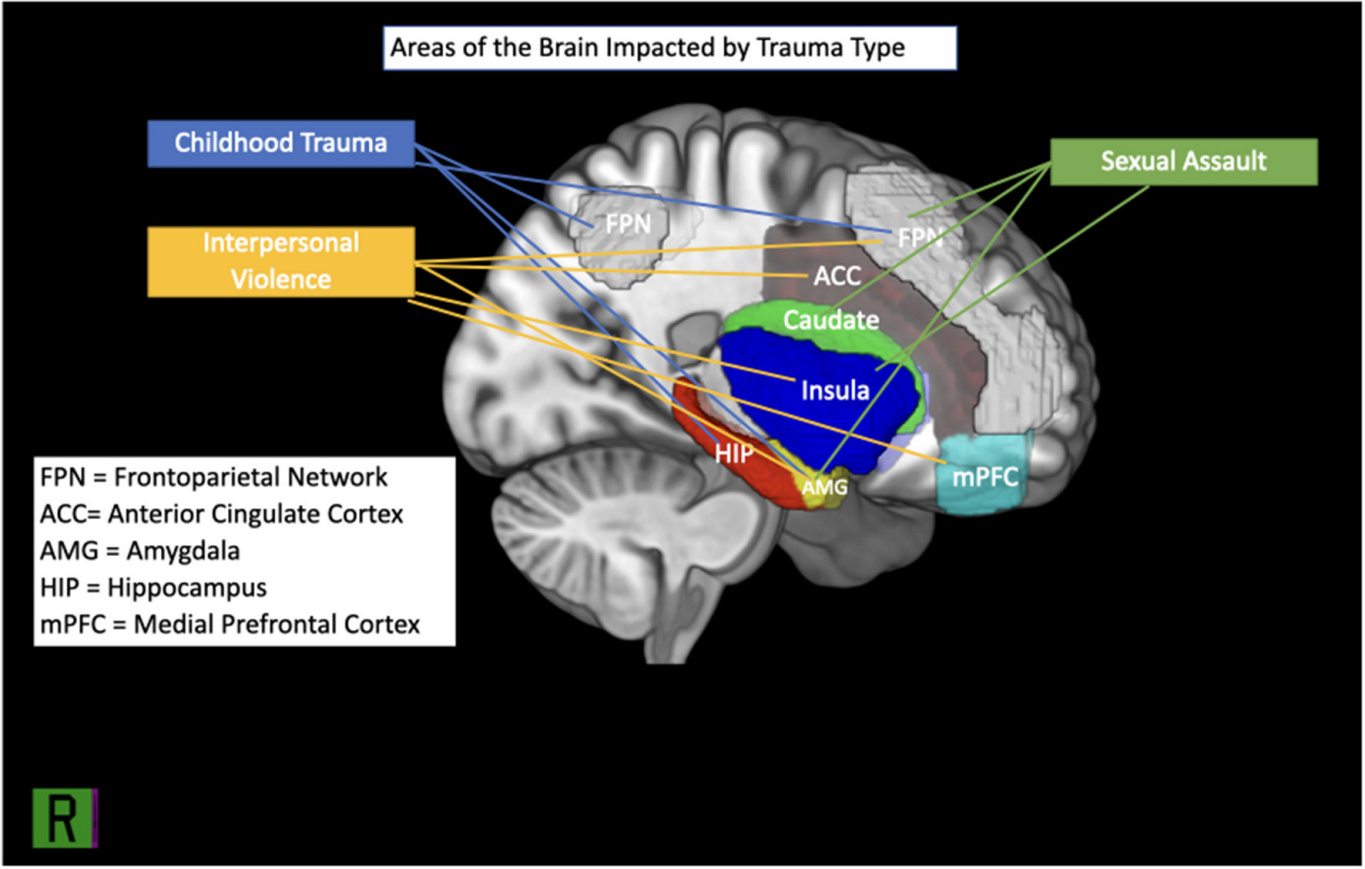
Brain regions implicated in IPV, childhood trauma and sexual assault.

Neurodevelopmental outcomes of female victims of CSA with PTSD appear to differ from those of healthy controls. Areas of activation in the brain associated with CSA can be seen in Figure 1. In adolescence, female victims of CSA showed compromised BOLD activity using reinforcement learning fMRI tasks showed compromised activity and increased cortical thickness within the frontoparietal network (FPN) (68, 69, 73). As survivors develop into adulthood, such impacts to the FPN may translate into compromised neurological and cognitive functioning (63, 74).

Adult female victims of CSA that occurred before the age of twelve may exhibit decreased gray matter volume (GMV) in the visual cortex and right basolateral and cortical amygdala, in addition to smaller splenium than healthy controls (63, 75). Similarly, they continue to exhibit alterations in the FPN and somatosensory network sizes in resting state fMRI tasks, which vary according to symptom severity (59, 68). We interpret these findings of the protraction of deficits in the FPN, together with reduced GMV in the amygdala and visual cortex, to suggest that female adult survivors of CSA may struggle with aspects of executive functioning, including visual memory, attention shifting, and learning.

The severity of these deficits is likely influenced by the timing of CSA. For example, the literature has established that sexual abuse between 10 and 11 years is likely to damage the amygdala (59). Other critical periods have been established for the hippocampus (3–5 years), corpus callosum (9–10 years), and the prefrontal cortex (14–16 years) (4). The ways that these neurobiological presentations manifest as clinical symptoms of PTSD will be discussed further in a following section of the review.

Additional literature about childhood maltreatment and abuse pertains to samples of adolescents and adult females with mixed types and histories of physical, emotional, and sexual abuse in childhood, which we will refer to as childhood maltreatment. There is expanding evidence to support the notion that female adolescent brain development is affected by childhood maltreatment. For example, in adolescent victims of mixed types of childhood abuse, brain volumes are compromised in the insula, splenium, hippocampal, and frontal lobe suggesting an increased rate of myelination in comparison with healthy controls (67). This may lead to alterations in executive functioning and processing of self-referential information (5, 30). The deviation from normative development in the splenium, otherwise one of the fastest-developing parts of the corpus callosum in normative samples between 4 and 18, can debilitate visual focus and the modulation of visual information in the thalamus (76). In all, these varied pathways of neurological growth due to childhood abuse have the potential to impact development across the lifespan.

As females develop into adulthood, these deficits appear to continue throughout the brain, manifesting as overall reduced GMV and cortical thickness (CT) in some areas (i.e., the amygdala, visual cortex, midcingulate cortex, subiculum and presubiculum; see Figure 1) and increased CT in others (i.e., FPN) (5, 59, 63, 64, 69, 72, 74). Resting state functional connectivity appears interrupted between the visual, frontoparietal, and default mode networks (59). Similarly, adult females with a history of childhood abuse show more activity in the amygdala in response to fearful stimuli than healthy controls, which may suggest challenges in top-down regulation (49). In some female survivors of childhood maltreatment, such neurobiological profiles may result in challenges with executive functioning, self-referential processing, social relationships, and emotional regulation [e.g., (5, 57, 67)].

As with CSA, the nature and extent of the neurobiological and subsequent psychological impact of childhood maltreatment appears to be associated with its severity and duration (59, 67, 69, 72–74). For example, dissociation severity has been negatively correlated with GMV in the ventral attention network (VAN) (59). The duration of childhood maltreatment and CSA and corresponding severity of neurological alterations in males and females is also likely a combination of biological and psychosocial considerations. For example, if a child feels safe disclosing abuse to a caregiver then the continuance of the abuse might be less. In such cases, the child's social environment would therefore have facilitated a lesser duration of abuse, perhaps leading to improved neurobiological outcomes (77). This again underscores the probable relationship between environment and biology in the development of PTSD and potential neurological impact (77). Further neuroimaging research is needed to examine the specific interaction of nonbiological and biological factors on neurobiological outcomes of childhood maltreatment and sexual abuse in females.

##### Trauma Timing Summary

Female survivors of CSA and other forms of childhood maltreatment have distinct neurobiological profiles when compared to healthy controls, with modified aging in various parts of the brain associated with emotion regulation, executive functioning, and learning. Specifically, the literature accentuates differences in the connectivity and cortical thickness of FPNs between healthy controls and survivors of sexual abuse. Concurrently, it indicates varying outcomes in GMV in the visual cortex, frontal lobe, corpus callosum, and the VAN in survivors of other types of childhood maltreatment. Because much of the literature on childhood maltreatment works with samples who have suffered different types of abuse, it is likely that some of the results in the literature pertaining to general childhood abuse overlap with those specifically examining CSA. Type of abuse notwithstanding, the impact of the abuse on brain development is directly correlated with the age at which the abuse occurred, its severity, and duration (4, 66). The literature also highlights the distinct severity of the neurobiological impact of abuse prior to the age of 12 [e.g., (63)], thereby providing support for critical periods of neurological development in females. The extent of the modifications in neurobiological presentations resulting from abuse is likely also a function of environmental and social factors.

#### Trauma Type

Females are more vulnerable to interpersonal violence than males (78, 79). Broadly, interpersonal violence is emotional, physical, or sexual violence by a perpetrator who may or may not be an intimate partner. The literature describes two types of interpersonal violence that are more commonly directed toward females: intimate partner violence (IPV) and sexual assault. IPV is defined as physical, sexual, or emotional violence at the hands of a romantic partner or significant other (78, 80). In the literature, sexual assault is also examined without specification as to whether the perpetrator is an intimate partner. Other studies investigate interpersonal violence using samples that combine types of interpersonal violence (e.g., physical, sexual, emotional abuse). Therefore, a neural typology of specific types of interpersonal violence is difficult to ascertain. We will explore the evidence for existing patterns in the following sections.

##### Intimate Partner Violence

Adult females are more likely than males to experience and report being victims of intimate partner violence in their lifetime than males (78), yet only eight of the neuroimaging studies reviewed specifically address neural correlates of IPV. Although scarce, existing literature suggests that female survivors of IPV with PTSD exhibit neurobiological changes relative to healthy controls. Aberrations from healthy controls manifest in various ways across the frontoparietal, default, and salience networks, and translate into difficulty with executive functioning, emotional regulation, and hypervigilance [e.g., (29, 81)].

Within-group comparisons suggest divergence among females with IPV-PTSD from healthy controls in activity in the amygdala, anterior cingulate cortex, insula, dorsolateral prefrontal cortex, and medial prefrontal cortex (21, 29, 82). In general, it appears that some female survivors of IPV may struggle to accurately appraise situations as threatening or not and, in turn, to adjust neural responses to task demand (29, 82). Challenges with top-down regulation are reflected in trends toward increased activity in the medial and dorsolateral PFC, anterior ACC, and insula evaluating social and emotional stimuli in comparison to healthy controls (21, 29, 35, 82–84). Conversely, a proclivity toward hypoactivation of the frontal and parietal lobes when neural activation is required suggest further challenges modulating arousal, perhaps leading to avoidance and numbness often reported in PTSD (29, 50, 82). In some females, such issues may be further compounded by hyperactivity in the anterior cingulate cortex and dorsolateral prefrontal cortex, suggesting deviations from normative abilities to focus and integrate contextual information into emotional responses (35). Some female survivors of IPV-PTSD exhibit disorganized connectivity between the frontal and limbic systems, resulting in sustained periods of heightened arousal that may cause them to misdirect and fixate their attention on nonthreatening stimuli (82).

Taken together, these deficits may leave females with PTSD vulnerable to further neural dysregulation in the face of future trauma. Clinical presentations of PTSD in female survivors of PTSD may also be impacted by social and environmental variables, such as lack of perceived social support and cultural messages about blame in intimate partner violence (10, 81). The magnitude of the effect of these and other environmental and social covariates on neurobiological presentations in female survivors of IPV remains to be explored in the neuroimaging literature examined for the present review.

##### Sexual Assault

Adult females are more vulnerable to SA than males (79), making the neurobiological profiles of female survivors of SA with PTSD of particular interest. Of the studies reviewed, five directly examined survivors of sexual assault. Similar to IPV survivors, the female samples of sexual assault presented with varying alterations of GMV, activity and functional connectivity in the brain areas frequently implicated in PTSD (e.g., the insula, amygdala, right caudate, prefrontal cortex, and medial occipital cortex). The composite of the neural correlates found in the literature suggest hyperactivity in the insula and prefrontal cortex, in addition to aberrant GMV in the amygdala, right caudate, and insula in female survivors of SA as compared to healthy controls (22, 23). Similar to IPV survivors, the female samples of SA survivors also presented with changing degrees of activity and resting-state functional connectivity (rsFC) in the brain areas frequently implicated in PTSD (e.g., the insula, amygdala, right caudate, and medial occipital cortex) (22, 23, 34). Hypoactivation in the prefrontal cortex provides further support for challenges with emotional regulation and the executive functioning required for attention shifting (39). Collectively, female survivors of SA who have PTSD may face greater difficulties in the realms of information processing related to self and others, hyperarousal, and executive functioning than healthy controls. As a result, they may be more susceptible to PTSD symptoms of greater severity if faced with subsequent traumatic events.

Given the paucity of studies specifically examining the neural correlates of IPV-PTSD due to sexual assault in female samples, it is difficult to determine how females with IPV and sexual assault might be distinct in their neurobiological presentation. Results suggest similar presentations wherein there may be compromised ability to manage arousal in the amygdala and insula, thereby engendering deficits in automatic emotional processing. Such alterations may necessitate greater activation of the prefrontal cortex, perhaps reflecting the brain's attempt to overcompensate for a lack of control in the amygdala and insula. Relative to healthy controls, differences in social processing are fairly common in female survivors of sexual abuse, perhaps due to smaller volumes of the right caudate. There is also research to suggest that there are sociocultural elements that may predispose females to sexual assault, such as living in poverty and prior history of abuse (85). Additional research is needed to determine whether there is a difference in neural underpinnings of sexual assault and IPV, and the ways that society and culture may influence outcomes and neurobiological symptoms of PTSD in female survivors of sexual assault and IPV.

##### Mixed Samples of Interpersonal Violence

Remaining studies addressing interpersonal violence in females combine types of interpersonal violence, diluting the impact of each type of interpersonal violence on the female brain. It is therefore difficult to establish a unique neurobiological profile for female survivors of interpersonal violence with PTSD based on extant literature. However, results echo those discussed in prior sections, with deviations from healthy controls in GMV in addition to variations in subcortical activity and rsFC. Different findings from those already reported provide insight into the role of the corpus callosum in the brain, specifically the genu (31). Others have found increased right caudate volumes, perhaps leading to difficulty managing social expectations and threat appraisal (26, 27). Increased GMV in this region of the brain have been associated with other forms of psychopathology, including psychosis (86), further emphasizing the degree of compromised functioning in females with PTSD. Because most are studies examining fMRIs related to specific symptoms of PTSD, they will be reviewed in further detail when we examine clinical presentations of PTSD and their neural correlates.

##### Other Trauma Types

There is a gap in the neuroimaging literature surrounding the neural correlates of PTSD in females related to types of trauma other than those described above (i.e., medical trauma, natural disasters, or other). Our literature search rendered only three neuroimaging studies examining the neural correlates of PTSD stemming from other types of trauma in females meet our inclusion criteria. One examines the neural correlates of PTSD among breast cancer survivors, finding no significant differences in amygdala and hippocampal brain volume as compared to healthy controls (87). One did not clearly specify the types of trauma that the females in their samples had experienced, and another mixed trauma from accidents with interpersonal violence. The results of these studies are discussed in later sections in which we identify neural correlates of specific symptom clusters of PTSD in females.

##### Trauma Type Summary

Although it is difficult to determine the association between the type of interpersonal violence females incur and neurobiological deficits in functioning, the literature appears to establish that interpersonal violence impacts females' brains (see Figure 1). More specifically, it seems to cause weakened regulation of arousal by impacting GMV in the amygdala and insula, while creating a deficit in rsFC between the insula, amygdala, and prefrontal cortex that weakens executive, cognitive, and social functioning. What remains unknown is the extent to which this impact is influenced by individual differences within samples, such as variability among subjects' trauma load, symptom severity. Further, study designs are largely cross-sectional and sample sizes are generally small, thereby reducing the generalizability of the results found. Finally, as we highlighted in each of the sections above, the extent of the interaction between trauma exposure and sociocultural factors on neurobiological presentations in females with and without PTSD remains largely unexplored by neuroimaging research.

### Underlying Purported Mechanisms

In addition to differential susceptibility to specific types of gendered trauma, there are individual determinants that influence neurobiological presentations in females with PTSD. In the present section, we will examine some of these, including the neuroendocrine system, coping mechanisms, and behavioral mechanisms of PTSD in females.

#### The Neuroendocrine System

The impact of the neuroendocrine system on fear circuitry in the brain is well-documented among healthy females. Specifically, due to the high amount of estrogen and progesterone receptors in the amygdala and hippocampus, these areas are particularly susceptible to differential functioning and volume throughout the female menstrual cycle (8). These fluctuations can manifest as more frequent and severe symptoms of reexperiencing intrusive memories, negative alterations in mood (e.g., increased anxiety and depression), and difficulty with memory and executive functioning. For example, extinction recall is negatively impacted during the early luteal phase of the menstrual cycle (8, 88).

Despite the abundant evidence that the neuroendocrine system influences individual PTSD symptoms in female humans and rodents, only one study met our inclusion criteria of a diagnosis of PTSD. Sartin-Tarm et al. (43) examined the impact of estradiol on fear habituation in females with and without PTSD and found that estradiol was positively correlated with activation in the anterior cingulate cortex and dorsomedial prefrontal cortex during fear habituation responses. Low levels of estradiol negatively predicted the ability to habituate to fear extinction among those with PTSD. These results further stress the importance of the timing of the traumatic event on neurological susceptibility to trauma responses and the development of PTSD.

#### Emotion Regulation and Gendered Coping Mechanisms

Challenges with emotional regulation among females with PTSD reflect changes in several regions of the brain, presumably impacted by posttraumatic stress. The neuroimaging literature posits that females' implicit and explicit emotional regulation is affected by altered neural connectivity. Broadly, it suggests hypoactivity in the vmPFC and hyperactivity in the amygdala (49, 89). This hyperactivity may result in excess physiological and emotional arousal in females with PTSD and, in turn, lead to deficits in executive functioning required for emotion regulation. This is particularly true when females diagnosed with PTSD are faced with tasks of increasing cognitive demand or emotional load (21, 39, 52, 89).

Emotion regulation is also affected by the positive or negative evaluation of stimuli and its relevance to the initial traumatic event. Among females with PTSD, more threatening stimuli are associated with increased activation in the hippocampus and amygdala and decreased connectivity between the insula, the dACC, and the amygdala relative to healthy controls (29, 53). Challenges with emotional regulation are also evident in bottom-up regulation wherein females show hypoactivity of the vmPFC in response to positive stimuli (56). Taken together, it appears that females with PTSD exhibit distinct modifications in neurobiology that result in greater emotional dysregulation relative to healthy controls, often as a function of cognitive load. These neurobiological alterations may interfere with the executive functioning required for emotion regulation (21, 33). Overall, these patterns in the literature reflect a neural hypoactivity when faced with positive events, and hyperactivity and an attentional bias when faced with negative events. It is likely that the negative attention bias, together with the gendered coping mechanisms previously discussed (i.e., rumination and neuroticism) contribute to the other symptoms of hyperarousal and create a unique profile of arousal in females with PTSD.

Some of the challenges with emotional regulation discussed may be related to socialization to more gendered coping styles and, in turn, impact neurobiological presentations in females with PTSD. Certain emotion-focused coping styles and personality traits may leave females more vulnerable to developing psychopathology when faced with stressors or traumatic events (15). Perhaps two of those most documented are rumination, relevant to the PTSD symptom of reexperiencing (90), and neuroticism, a personality trait related to general psychopathology at large (15, 16).

##### Rumination

Researchers have found that females ruminate more than men and that rumination, an emotion-focused coping style defined as self-referential thought focused on negative outcomes, is a transdiagnostic consideration in psychopathology (15). The tendency to ruminate has been correlated with sociocultural considerations such as identification with femininity, as well as biological sex factors like estradiol levels (12, 91). As the other constructs reviewed, rumination and its impact on PTSD symptoms is therefore likely the product of nature and nurture.

Extant literature characterizes rumination as the function of potential deficits with executive functioning, including memory (92), attentional shift (16), emotional processing, and coping (15). These correlate with hyperconnectivity between the right amygdala and the mPFC, PCC, precuneus, and the orbital cortex, reflecting the need to make a greater cognitive effort to shift attention sets (21). Conjointly, such weaknesses may cause those who ruminate to fixate on negatively-valenced emotional information, thereby leading to exacerbated outcomes among females with PTSD. For example, rumination in females with PTSD has been positively correlated with the frequency and intensity of reexperiencing symptoms, corresponding to increased GMV in the left isthmus cingulate (40, 90, 93).

##### Neuroticism

Much like rumination, neuroticism, a personality construct defined as one's proclivity toward experiencing negative emotional states, is associated with poor psychological outcomes (16, 94). Those who endorse higher levels of neuroticism may experience high levels of anxiety, have difficulty coping with challenging situations, and display poorer emotion regulation than others 16). Females with PTSD who display more neurotic tendencies experience PTSD symptoms of greater severity (95), making it important to understand the neural correlates of neuroticism.

Like rumination, neuroticism interferes with the ability to objectively evaluate environmental stimuli, due to an overactivation of brain regions associated with fear, self-referential processing, and value judgments needed to make accurate decisions. Neuroticism is positively correlated with hyperactivation in the amygdala, right PFC, dmPFC, and parahippocampus (16). Together, rumination and neuroticism leave females with PTSD more vulnerable to reexperiencing, negative alterations in mood, difficulty with memory, and accurate fear-based learning.

#### Behavioral Mechanisms

Behavioral mechanisms of PTSD involve fear-based learning, such as extinction, value expectation, and inhibition. Fear extinction is an important part of PTSD symptom reduction and recovery and is therefore a large part of APA-recommended PTSD treatments like exposure therapy (96, 97). Fear extinction requires the ability to accurately assess potentially fearful future events, to inhibit fear responses when they arise and decrease arousal, and the eventual ability to learn from experiences (98). The neuroimaging literature suggests that females diagnosed with PTSD present with deficits in the neural underpinnings of each of these processes, contributing to rigid thinking and learning styles which thereby hinder their recovery (21).

Accurate assessment of future events requires application of prior knowledge and experiences to future situations and their possible outcomes. The literature suggests that many females with PTSD may struggle to integrate prior experiences into their expectations and responses to situations and tasks at hand, in threatening and nonthreatening situations (42). Because females with PTSD may have difficulty incorporating lessons learned into decision making and problem solving, they may resort more to trial and error in problem solving tasks (68). This may be due to a propensity to prediction errors, combined with an attentional bias in the brain toward negative information that is consistent with inaccurate value expectations (26, 48). Females with PTSD are more neurologically reactive to anticipated negative events and less so to anticipated positive events than healthy controls, thereby further complicating the formation of expectations (47). Neuroimaging results suggest that these outcomes are associated with deficits in the FPN, and hypoconnectivity in the ACC and dmPFC (26, 68).

Inhibition is also an important part of reversing fear-based learning, involving the ability to monitor one's own mental and physiological state and to purposefully react to the same (21, 99). The literature reviewed reflects the notion that females with PTSD may struggle with inhibition, such that they may find it difficult to regulate arousal in response to external stimuli. These challenges are reflected in hyperactivation of the insula, dmPFC, the striatum, the amygdala, and hypoactivation of the vmPFC (21, 33, 48). Such abnormalities may result in difficulties with cognitive control, attention shifting, and processing of self-referential information, all processes required to inhibit arousal (47, 99). Such deviations from normative social learning, expectation formation, and inhibition may contribute to the documented mixed outcomes of exposure therapy in among females with PTSD.

Successful extinction has been negatively correlated with activation in the vmPFC and bilateral amygdala (96). However, females with PTSD display the opposite neurological profile when engaging in extinction learning tasks. Another important factor that is not often considered by the neuroimaging literature is the impact of individual estradiol levels on extinction learning, frequently overlooked by extant neuroimaging evidence (43). Although previously discussed, it is important to highlight it here as a factor that may make females more vulnerable to varying results in behavioral treatments of PTSD.

#### Underlying Mechanisms Summary

Females have distinct coping styles and ways of regulating their emotions, corresponding to personality (e.g., neuroticism), cognition, (e.g., rumination), and behavior (e.g., fear processing). These are each associated with a specific neural presentation and may be linked to both gender and sex. Although each has specific neural correlates, they appear to reflect a common pattern of decreased connectivity between the insula and dmPFC. In addition, the literature reflects patterns of hyperactivity in the amygdala, insula, dmPFC, and striatum with deficits in connectivity between the amygdala and the vmPFC. The mechanisms reviewed may leave females more vulnerable to worsening trauma symptoms and unique presentations of PTSD and comorbid disorders. In the following section, we examine the literature surrounding neural correlates of clinical presentations of PTSD in females, including symptom types, severity, and comorbidity.

### Presentation

The DSM-5 classifies PTSD according to criteria across four symptom clusters: reexperiencing, or experiencing unwanted memories of the traumatic event; hyperarousal, evidenced by symptoms such as being startled easily or hypervigilance; avoidance of stimuli that are reminders of the traumatic event; and increased negative affect across several domains, including irritability, anhedonia, and feelings of alienation from others (100). As previously noted, these symptoms often differ in severity and duration, often according to the type and timing of trauma. For females, clinical and neurobiological presentations may also be a function of the phase of their menstrual cycle in which the trauma occurs, together with age (8). In the present section we will review the different types of clinical presentations that arise in females with PTSD, according to symptom cluster.

#### Reexperiencing

A hallmark of PTSD is reexperiencing the traumatic event through intrusive thoughts, memories, or nightmares, which may be triggered by direct or indirect reminders of the traumatic event (100). The neuroimaging literature on females with PTSD examines reexperiencing through fMRI imaging of subjects being exposed to direct or indirect reminders of the traumatic experience [e.g., (35)], or through the correlation of self-report of reexperiencing with GMV and rsFC [e.g., (27)]. In general, the evidence suggests that females who indicate higher rates of reexperiencing symptoms exhibit altered neural correlates associated with visual memory and inhibition, and increased activity between the limbic and default networks.

Among females with PTSD, reexperiencing is associated with reduced CT in the left inferior and mid temporal gyrus and increased GMV in the lingual gyrus (22, 27). Diffusion Tensor Imaging (DTI) similarly suggests compromised white matter volumes (WMV) of the postcentral gyrus in the corpus callosum. The alterations of WMV in the corpus callosum may suggest deficits in the processing and communication of visual information between hemispheres (21, 31). The increased GMV of the lingual gyrus, also associated with inhibition and top-down regulation, may lead to difficulties in preventing memories from surging to conscious awareness. This may lead females with PTSD to feel flooded by visual memories of their traumatic event, thereby increasing overall arousal (101).

Distress associated with reminders of traumatic events is reflected in an increased rsFC between the right and left amygdala, the right inferior frontal gyrus, the right hippocampus, and the visual and dorsomedial cortex (27, 37, 38, 102). It is concurrently associated with hypoconnectivity between the lingual cluster and the fusiform cortex, responsible for the voluntary and involuntary processing of trauma memories (22). The combination of hyper- and hypoactivity in these areas of the brain may reflect the brain's altered ability to assimilate traumatic memories and prevent hyperarousal when confronted with reminders of the same.

#### Hyperarousal

Hyperarousal among individuals with PTSD manifests as irritability, impulsivity, hypervigilance, difficulty sleeping, inability to concentrate, and exaggerated startle responses (102). These symptoms may indicate deficits in emotion regulation, often described in PTSD as part of a “feedback loop” that leads to further arousal, thereby creating challenges with concentration and reliable evaluation of environmental stimuli (101).

Hypervigilance can generally be defined as a sense of constant awareness of one's surroundings, even in situations one knows to be safe. Surprisingly, we found the neural correlates of hypervigilance to be largely understudied in females with PTSD. However, the little evidence that exists describes a pattern of hyperconnectivity between the amygdala, insula, ACC and the superior colliculus and locus cereleus in females with PTSD (102). These areas of the brain, responsible for visual and auditory processing and arousal, suggest overactivation which, together with the negative attention bias previously discussed, may lead to appraisals of otherwise neutral stimuli as threatening (21, 102).

#### Negative Affect

Negative affect and changes in mood are often reflected in self-blame, exaggerated negative perceptions of self and others, anhedonia, and detachment or estrangement from others (102). Extant literature reports that many females with PTSD have an overwhelmingly negative self-image coupled with tremendous guilt relative to those without PTSD. They may find themselves debilitated in their ability to modify such negative cognitive distortions, due to rigidity in thinking, which has been connected to poor psychological outcomes (48, 57). Neural correlates reflect a greater preoccupation with the self than others as well as a negative attention bias toward their own qualities, relative to healthy controls. This attention bias may manifest as a tendency to accept more negative qualities about themselves, while also endorsing more positive qualities about others. Examinations of neural activity suggest greater activity of the visual cortex when imagining the self as opposed to others, in addition to greater activation in the mPFC when considering negative qualities about themselves (57). The correlates of self-blame mirror those of reexperiencing, exhibiting reduced GMV in the lingual gyrus, such that self-blame may involve an aspect of reexperiencing among females with PTSD (21, 57). Cumulatively, these results may suggest difficulty regulating emotions in relation to the self and negative cognitive distortions about the self, with favorable biases toward others.

#### Avoidance

Among males and females, avoidance is associated with heightened global PTSD symptom severity (103). Females with PTSD who engage in avoidance with greater frequency show reduced CT in the occipital gyri, as well as the left lateral fissure and right posterior cingulate (27). The posterior cingulate cortex has been found to mediate information between emotions and memory (104), while the lateral fissure and occipital gyri have been associated with visual and working memory. The reduction in CT may suggest a decreased capacity to process emotional memories and uncertainty about the safety of their surroundings due to prediction errors. In turn, individuals with PTSD may develop avoidance as a coping skill to escape overwhelming feelings that may be associated with their inability to accurately process emotional and environmental stimuli.

#### Specifiers: PTSD With Depersonalization or Derealization

The most recent diagnostic classification for PTSD includes a specifier for a dissociative subtype (PTSD-D) to identify the presence of persistent symptoms of depersonalization or derealization. DSM-5 defines depersonalization as “persistent or recurrent experiences of feeling detached from, and as if one were an outside observer of, one's mental processes or body,” and derealization as “persistent or recurrent experiences of unreality of surroundings” (105). PTSD-D can therefore be summarized as a presentation of PTSD in which there is a disconnection, or dissociation, from one's lived experience.

Extant neuroimaging literature comparing females with PTSD-D to healthy controls suggests that dissociative experiences relate to hyperactivity in the somatomotor network (i.e., the right superior temporal gyrus, involved in auditory processing) and frontoparietal lobes, specifically in the left inferior, precentral, and medial gyri (59, 60). Higher rates of dissociation severity are negatively related to activation in the parahippocampal gyrus, associated with memory formation and spatial location of objects, and hypoactivity in the ventromedial prefrontal cortex (59, 60, 106). Accordingly, the neurobiological profile of females with PTSD-D suggests difficulties with emotion regulation, likely caused by a sense of sensory and emotional overwhelm. Altered activity in the vmPFC may suggest deficits in amygdala regulation (89), leading to challenges in downregulating excessive arousal, and hypervigilance among some females with PTSD. This presentation is heightened by deficits in synchronization between key brain regions including the amygdala, hippocampus, thalamus, and the brain stem (61), proposed to be linked to the experience of dissociation.

#### Presentation Summary

Females with PTSD present with altered GMV and WMV in areas of the brain responsible for emotion and visual processing. These deviations from volumes in healthy controls correspond to hypoactivity of brain structures involved in top-down and bottom-up emotion regulation (i.e., the vmPFC) and executive functioning (i.e., the dmPFC), in addition to hyperactivity of areas related to arousal (i.e., the amygdala) and sensory processing (i.e., the right superior temporal gyri).

#### Comorbidities

A review of clinical presentations of PTSD in females would be incomplete without a discussion of the literature related to comorbidities. As many as 83% of males and females diagnosed with PTSD present with a comorbid disorder, and female gender has been identified as a risk factor for the same (107, 108). As such, it is important to review the neuroimaging literature regarding specific comorbidities that occur with PTSD. PTSD rarely occurs by itself, and it is frequently associated with comorbidities such as Major Depressive Disorder (MDD), Dissociative Identity Disorder (DID), Borderline Personality Disorder (BPD), and Substance Abuse Disorder (55, 109, 110). Past literature has studied neural correlates of each of the aforementioned comorbidities with PTSD in females except Substance Abuse Disorder.

##### PTSD and Major Depressive Disorder

Reports on the incidence of PTSD-MDD vary, but the literature reports it as one of the most commonly occurring comorbidities among patient populations with PTSD. Recent statistics show that up to 89% of males and females with PTSD present with PTSD-MDD (108–110). Despite the high prevalence of depression among PTSD populations, there is little neuroimaging research that specifically investigates the neural correlates of PTSD-MDD in females. The only study we found that met the inclusion criteria for this review implicated a hypoconnectivity between the amygdala and the frontal and temporal lobes in PTSD-MDD (44). These results replicate others found in literature that examined PTSD, perhaps because so many studies contain samples with mixed presentations of PTSD in females, including PTSD-MDD [e.g., (49)]. Despite these findings, there is still not enough information to clearly establish a neurobiological profile for PTSD-MDD.

##### PTSD and Borderline Personality Disorder

A comparison of the symptoms of BPD and PTSD in the DSM-5 reveal an overlap of symptoms. Patients of BPD and PTSD both present with difficulty with emotion regulation; an unstable sense of self, marked by negative self-image; dissociative symptoms; and a proclivity toward impulsivity or behaviors that might incur self-harm (100). As such, one might expect that the literature would reflect similarly impacted areas in the brain, both in GMV and rsFC, perhaps on a larger scale due to the presence of a dual diagnosis. However, the study that met the inclusion criteria for the present review actually found no significant differences between GMV of the amygdala, vmPFC, or bilateral ACC when comparing females with BPD and PTSD to those with PTSD alone (51). The dearth of neuroimaging literature surrounding BPD and comorbid PTSD, en masse with these results that suggest insignificant differences between neurobiological profiles, suggests the need for further investigation of this comorbidity.

##### PTSD and Dissociative Identity Disorder

PTSD-DID has been examined largely as it pertains to differences in morphology between females afflicted with the disorder and healthy controls. Results are again insufficient in volume to reveal a specific neurobiological profile of PTSD-DID, but portray interesting findings surrounding subcortical GMV in females with PTSD-DID. More specifically, PTSD-DID has been associated with reduced hippocampal GMV with concurrent increased GMV in the right putamen, pallidum, and striatum as a function of the severity of dissociative symptoms (5, 55). It is unclear from the current body of literature whether these neural correlates also correspond to those with PTSD-D.

##### Summary of Comorbidities

In general, there is a lack of information surrounding comorbidities with PTSD and associated neural correlates in female samples that precludes conclusions about specific neurobiological profiles of each. Perhaps one of the most challenging components of studying such comorbidities is that PTSD symptoms are shared among so many psychiatric disorders, such that determining specific neural correlates is particularly challenging (44). It is perhaps this overlap in symptoms and variety of presentations that lead to differing outcomes and prognoses among females with PTSD.

### Sex Related Outcomes of PTSD

We turn now to an examination of the outcomes of PTSD among females, looking at some of the ways that trauma further impacts them specifically. We begin by reviewing literature on the ways that PTSD impacts mothering and corresponding neural correlates and end with a review of treatment outcomes.

#### Parenting Outcomes: Mothers With PTSD and Their Children

Children of mothers with PTSD are more likely to experience traumatic events than children of healthy mothers (111). The literature documents one possible explanation as lower levels of attunement and maternal sensitivity among mothers with PTSD, particularly if a mother endured severe childhood trauma [e.g., (45, 112)]. Parenting requires the ability to mentalize (i.e., take another's perspective by identifying their emotions), empathize, and respond sensitively to a child's needs (36). As we have documented, PTSD may result in emotional dysregulation and a proclivity toward interpreting neutral social information as threatening, resulting in a state of persistent hyperarousal. In mothers, it is likely that such constant hyperarousal complicates their ability to respond sensitively to their young.

Evidence for heightened arousal when faced with stress in relation to their young can be found in decreased activation of the vmPFC among mothers with PTSD relative to healthy controls (36, 46). Altered emotion regulation may cause mothers with PTSD to respond differently to separation from their children than healthy controls, manifesting as heightened arousal and increased activity in the dmPFC (36). Thus, mothers with PTSD may have more difficulty engaging in parental reflective functioning relative to healthy controls, thereby complicating the formation of a secure attachment with their children.

Children of mothers who suffered childhood abuse exhibit patterns that mirror their mothers' emotion regulation, evidenced by decreased vmPFC activity. Relative to children of healthy controls, the literature points to a higher probability that children of mothers with PTSD endure a traumatic event or develop psychopathology, perhaps related to emotion regulation and arousal (46, 111). Together, the neuroimaging literature reflects the detrimental impacts of intergenerational trauma on youth when a mother has PTSD, making it imperative that the field move toward a greater understanding of the etiology, presentation, and treatment of PTSD in females.

#### Treatment Outcomes

Results of clinical trials of treatment in females with PTSD suggest that outcomes are largely dependent upon trauma severity, independent of age or clinical approach. Few approaches have been examined solely in female samples, emphasizing the challenge in coming to conclusions regarding best practices specifically for females with PTSD. No treatment was reviewed more than once in the literature reviewed, such that results have not been replicated among more than one sample of females.

Extant neuroimaging literature investigates the impact of Trauma-Focused Cognitive Behavioral Therapy (TF-CBT), Exposure Therapy (ET), and Cognitive Processing Therapy (CPT) on rsFC (20, 21, 25, 65). Results suggest that, regardless of the modality, effective treatments improve connectivity between the amygdala, the insula, the right hippocampus, and the right superior frontal gyrus (21, 25, 65). Therapy for PTSD among females has also been correlated with an increase in WMV in the splenium and left cingulate gyrus, thereby rehabilitating communication between the left and right hemispheres (32). Symptom reduction from treatment is therefore likely observed in increased emotional and physiological regulation, thereby granting patients a sense of greater self-control and reduced fear.

Such outcomes appear to be predicted by trauma type and severity more than other individual factors, such as age (20, 25). As previously established, these covariates moderate the degree to which neural correlates change as a result of trauma, and treatment outcomes are often a function of pretreatment neurobiology. For example, the success of TF-CBT appears to be a function of pre-existing amygdala connectivity with the dACC in threatening situations (65). Similarly, pretreatment levels of activation in the amygdala and the insula, corresponding to anticipation of negative events, predicted CPT outcomes (20). Outcomes in the corpus callosum are similarly predicted by the extent of the damage that existed pretreatment (32).

As a whole, there is evidence to suggest that current evidence-based treatments are efficacious in repairing certain neurological impacts of PTSD in females, particularly for those who present with mild to moderate trauma loads. However, little is known about meaningful interventions for victims of more severe types of trauma, highlighting an area for further research.

## Discussion

This review addresses the neurobiological presentation of females with PTSD and potential covariates that may contribute to the higher incidence of PTSD reported among females. The significance of the type and timing of the traumatic event, the neuroendocrine system, gendered coping styles, and outcomes of trauma in females are discussed. Where possible, we highlighted the likely relationship of biological and nonbiological factors on PTSD outcomes in females. We identify areas of further research pertaining to females with PTSD.

In general, females with PTSD present with altered GMV, WMV, and connectivity between the amygdala and prefrontal cortex. Females are more vulnerable to specific types of traumatic exposures than males, but based on the current body of research, it is difficult to establish a specific neurobiological profile in females with PTSD according to the type of traumatic event. Many of the studies examining interpersonal violence use samples with survivors of different types of interpersonal violence, complicating the ability to make clear conclusions. Prior literature underscores the impact of sexual assault on females, noting its potential to modify DNA expression (113, 114). Such results emphasize the need for further research that clearly distinguishes between trauma types in samples. Additionally, as with most neuroimaging literature, the generalizability of the results is limited by small sample sizes, convenience samples, mixed trauma histories within samples, and cross-sectional designs. Because neuroimaging studies largely do not focus on cultural factors affecting clinical outcomes, the findings presented do not adequately consider cultural factors that may influence brain development and outcomes in PTSD.

The present review summarized the impact of the type and timing of trauma on PTSD outcomes in females. Type of trauma notwithstanding, it appears that trauma is most detrimental when it occurs in childhood, modifying the naturally occurring aging process in the brain. In females, childhood trauma is associated with specific symptoms of PTSD, such as dissociation (5). The severity of these symptoms varies as a function of the duration and severity of abuse, which is likely related to social factors in the victim's environment. The susceptibility of victims of childhood abuse to adverse neurobiological outcomes is consistent with prior literature suggesting that there are critical periods for neurological development in which the brain is more vulnerable to the effects of stress and trauma (4, 83). The literature makes a distinction between childhood abuse before the age of 12, partially due to the onset of menstruation in females.

Our findings suggest that the type of trauma sustained is perhaps less influential on the brain than a victim's age upon initial trauma exposure. This may be due to the relative lack of comparison between trauma types among females in the neuroimaging literature. Nonetheless, there are unique factors to consider when examining the influence of trauma type on brain development that are not necessarily addressed by extant neuroimaging literature. For example, IPV is distinct from interpersonal violence because of the unique dynamic in which a victim knows and has a close, intimate relationship to their assailant (10). This experience is uniquely accompanied by betrayal trauma, in addition to specific subtleties related to disclosure and subsequent emotional and psychological impacts of the same (11). Such contextual factors create nuances in the experience of trauma exposure and type that are not reflected in the current body of literature, such that trauma type may be more influential on the brain than current neuroimaging findings suggest.

Another factor that was ostensibly absent from the neuroimaging literature examined in the present review was the female neuroendocrine system. Although the literature reviewed acknowledges the impact of menstrual phase on female presentations, it offers little insight regarding the effect of the neuroendocrine system on neurological and symptom presentation of PTSD in females. Other reviews have documented that the phase of the menstrual cycle correlates to memory, fear responses, reexperiencing, and negative mood in females with and without PTSD (8, 115). Similarly, the timing of trauma in relation to the onset of puberty has also been found to predict severity of PTSD symptoms (116). Consistent with other literature examining the impact of estradiol on learning, the present review found one study that suggested a positive association between estradiol and fear extinction (43). Female hormones of estradiol and progesterone fluctuate throughout the menstrual cycle, and this variation is moderated by the hippocampus (8). The hippocampus itself has been shown to fluctuate in volume throughout the menstrual cycle and has been associated with positive treatment outcomes [i.e., (117, 118)]. It would therefore seem logical that further research investigate the impact of the neuroendocrine system on females' presentation of PTSD and neurobiological substrates throughout the menstrual cycle. Because it is largely ignored by the neuroimaging literature, it is likely that the field is missing information necessary to establish a complete typology for females with PTSD. Put simply, it appears that hormonal fluctuations are a covariate that many authors have failed to consider in extant neuroimaging research on females diagnosed with PTSD.

However incomplete due to the lack of studies examining the neuroendocrine system, there is evidence that females engage in specific types of emotion-based coping that may, in turn, influence the severity of their neurological and psychological symptoms. Neuroticism and rumination may be rooted in societal beliefs that problem-oriented problem solving and coping is masculine (17, 40, 119, 120). Such coping styles and traits have been correlated to negative emotional outcomes which, when combined with PTSD, may leave females particularly vulnerable to developing further psychopathology. Neurobiological profiles associated with these traits highlight increased GMV in the temporal lobe and hyperactivation in the prefrontal cortex (17, 90). These alterations lead to more frequent incidences of reexperiencing and hyperarousal, thereby further debilitating already compromised emotion regulation and executive functioning in some females with PTSD (90). Such exacerbated presentations create mixed outcomes for females with PTSD, particularly as it pertains to maternity and treatment.

Females with PTSD may have more difficulty establishing secure attachments with their children than healthy controls (121). Although not exclusively focused on females, prior studies have found that dismissive and fearful attachment styles correlate with dismissive or fearful adult attachment styles (122). Mothers with dismissive attachment styles may be less sensitive to their children's needs, reflecting a deficit in mentalization and parental reflective functioning (36, 45, 46, 123). The neuroimaging literature suggests that these deficits are correlated with increased arousal and activity in the prefrontal cortex when faced with stress in the parent-child relationship. It is likely that cultural and societal factors are also important in parenting outcomes among females with PTSD, but this is largely unexplored in the present neuroimaging literature pertaining to mothers.

Although the presentation of symptoms for females with PTSD appears bleak and compounded by the unique types of traumatic events they may experience, there is reason for hope. Treatment outcomes suggest moderate efficacy among those with mild to moderate PTSD. Like the remaining body of literature, studies are limited by small sample sizes, mixed sources of trauma, comorbidities, and overlapping symptoms. Further research is needed to understand ways of reaching females with more severe symptoms of PTSD, in addition to the effect of cultural and societal factors and their interaction with neurobiology on symptom severity and prognosis.

### Limitations

Perhaps the most important limitation of our review is that a focus on neurobiological substrates of PTSD in females detracts from the worthwhile examination of societal and cultural factors influencing PTSD symptom presentation and severity in females. For example, gender norms often impact the way females manifest psychopathology [e.g., (14)]. While we attempted to address this shortcoming by discussing gender norms surrounding emotional coping styles, there are certainly other nonbiological factors that impact PTSD in females, such as perceived social support and peer reactions to trauma disclosure (10). We posit that this weakness is also a reflection of the biological focus of neuroimaging literature, and suggest that future neuroimaging research consider more thoroughly the impact of cultural and societal influences on the neurobiological presentation of females with PTSD.

By including only studies that used MRI imaging, we did not report on several studies that use PEG and EEG methods in female samples with PTSD that may have been relevant to our review, of which there are several. Including studies that used other forms of neuroimaging may have provided a more complete picture of females with PTSD, symptom clusters, and their neural correlates. Many MRI studies use convenience samples, such that the participants within the studies reviewed do not likely represent all females with PTSD. Most samples consisted of predominantly white females, thereby limiting the generalizability of the findings presented to females of color or those who are nonbinary.

By excluding studies in which subjects had probable instead of confirmed PTSD, we likely failed to include valuable information about females' presentation and recovery from PTSD. To that end, PTSD is not the only clinical outcome of a traumatic event as many females present with Major Depressive Disorder as their primary diagnosis. Such studies are equally important to the present body of literature, but were not included in this review for the sake of cohesiveness. Similarly, by including only studies that make comparisons of females with and without PTSD, we cannot specify whether the findings described here are sex-specific or sex-related. Findings in mixed sex samples point to sex similarities and differences in neural connectivity in PTSD. Findings in mixed sex samples point to sex similarities and differences in neural connectivity in PTSD. For example, both females and males with PTSD show increased activation in the dACC in tasks of extinction (124). On the other hand, Helpman et al. (125) found that females with PTSD displayed rs-FC patterns that were the opposite of males and healthy females, pointing to sex-specific neural patterns in PTSD. Such mixed findings underscore the need for further research examining females with and without PTSD. Finally, as the neuroimaging literature does not consistently report the gender identity of participants, the impact of gender, rather than sex, cannot be teased out. Moreover, the pertinence of the findings to persons across the gender spectrum may be limited. Further research is needed to address the relevance of findings to those across the gender spectrum, in addition to the replicability of prior outcomes.

To summarize the literature, we grouped samples according to trauma type and timing. In doing so, we may have overlooked the fact that many with PTSD experience multiple traumas in childhood and adulthood. This, however, is also reflected in the current body of literature that amalgamates trauma types and timings in an effort to comprehend the neural underpinnings of PTSD of females. Similarly mirroring the neuroimaging literature which often presents mixed findings, the neurobiological outcomes discussed may differ from those previously found by other authors. However, fMRI studies in particular are frequently scrutinized for limited replicability based on imaging methodology, ROI used, and fMRI tasks involved (126, 127). Therefore, these differences may also be the result of varying study designs and procedures. Finally, due to the amplitude of studies included and the varying methods they used, we synthesized information about some regions of the brain more broadly instead of referring to specific brain regions, such as the prefrontal cortex and anterior cingulate. Many studies examine different areas of each, such that results were grouped according to their larger brain region for clarity. We suggest that future reviews focus specifically on the impact of PTSD on these brain regions in females, thereby providing an opportunity for richer discussion of the workings of the prefrontal cortex and anterior cingulate cortex.

## Conclusion

The present review highlights biopsychosocial factors that may make females vulnerable to PTSD, clinical presentations of PTSD in females, and their neural correlates. Clinical presentations in females reflect deficits in the brain related to executive functioning (i.e., dmPFC), emotion regulation (i.e., amygdala), and inhibition (VAN). The differences between females with and without PTSD highlighted in this review reflect important considerations for future research, particularly as it pertains to the impact of the neuroendocrine system on clinical presentations of PTSD. Similarly, we highlight the need for more consistency in samples of females with PTSD, especially as it pertains to comorbidities, trauma type, and timing. We are hopeful that, in years to come, there will be more representation of females in PTSD literature such that their needs are more specifically considered by psychology and psychiatry.

## Data Availability Statement

The original contributions presented in the study are included in the article/supplementary material, further inquiries can be directed to the corresponding author.

## Author Contributions

EE-M: literature search, writing-original draft, and revisions. XZ and MB: writing-review and editing. CF: literature search and writing—editing. LH: conceptualization and writing-review and editing. YN: conceptualization, funding acquisition, supervision, and writing-review and editing. All authors contributed to the article and approved the submitted version.

## Funding

This research was supported by United States - Israel Binational Science Foundation (BSF) grant #2019137 (PIs LH, YN), Israel Science Foundation (ISF) grant #2107/17 (LH), NIMH grant R01MH105355-01A1 (YN-PI). XZ was supported by K01MH122774 and Brain & Behavior Research Foundation.

## Conflict of Interest

The authors declare that the research was conducted in the absence of any commercial or financial relationships that could be construed as a potential conflict of interest.

## Publisher's Note

All claims expressed in this article are solely those of the authors and do not necessarily represent those of their affiliated organizations, or those of the publisher, the editors and the reviewers. Any product that may be evaluated in this article, or claim that may be made by its manufacturer, is not guaranteed or endorsed by the publisher.
